# A Novel Dataset and Detection Method for Unmanned Aerial Vehicles Using an Improved YOLOv9 Algorithm

**DOI:** 10.3390/s24237512

**Published:** 2024-11-25

**Authors:** Depeng Gao, Jianlin Tang, Hongqi Li, Bingshu Wang, Jianlin Qiu, Shuxi Chen, Xiangxiang Mei

**Affiliations:** 1 School of Yonyou Digital and Intelligence, Nantong Institute of Technology, Nantong 226001, China; gaodepeng@ntit.edu.cn (D.G.); qiujl@ntit.edu.cn (J.Q.); chenshq@ntit.edu.cn (S.C.); meixiangxiang@ntit.edu.cn (X.M.); 2 School of Software, Northwestern Polytechnical University, Xi’an 710000, China; shibasakiakira@mail.nwpu.edu.cn (J.T.); lihongqi@nwpu.edu.cn (H.L.)

**Keywords:** anti-UAV detection, anti-interference anti-UAV dataset, YOLOv9

## Abstract

With the growing popularity of unmanned aerial vehicles (UAVs), their improper use is significantly disrupting society. Individuals and organizations have been continuously researching methods for detecting UAVs. However, most existing detection methods fail to account for the impact of similar flying objects, leading to weak anti-interference capabilities. In other words, when such objects appear in the image, the detector may mistakenly identify them as UAVs. Therefore, this study aims to enhance the anti-interference ability of UAV detectors by constructing an anti-interference dataset comprising 5062 images. In addition to UAVs, this dataset also contains three other types of flying objects that are visually similar to the UAV targets: planes, helicopters, and birds. This dataset can be used in model training to help detectors distinguish UAVs from these nontarget objects and thereby improve their anti-interference capabilities. Furthermore, we propose an anti-interference UAV detection method based on YOLOv9-C in which the dot distance is used as an evaluation index to assign positive and negative samples. This results in an increased number of positive samples, improving detector performance in the case of small targets. The comparison of experimental results shows that the developed method has better anti-interference performance than other algorithms. The detection method and dataset used to test the anti-interference capabilities in this study are expected to assist in the development and validation of related research methods.

## 1. Introduction

Unmanned aerial vehicles (UAVs) are valued for their flexibility and autonomy and have been widely used in photography, communications, cargo delivery, and remote sensing. However, the improper use of UAVs has seriously disrupted the normal social order [1,2,3]. For instance, in April 2018, London Gatwick Airport was forced to suspend all flights due to a UAV intrusion into its airspace, affecting more than 100,000 passengers. Despite the new opportunities for the economy and society arising from advancements in their application, UAVs still pose significant challenges for security and management.

Among the traditional anti-UAV technologies, radar detection has undoubtedly been one of the most prominent. However, with the continuous development and iteration of UAVs, they have decreased in overall size and are being flown at lower altitudes. In particular, their radar cross-section has been significantly reduced [4,5]. Due to the natural low-altitude blind area, traditional radar systems are proving ineffective for detecting the “low–slow–small” UAVs. Therefore, under the current conditions, traditional radar detection is no longer sufficiently effective for completing the basic task of anti-UAV detection.

In recent years, target detection models based on deep learning have seen considerable development and expansion, and the above drawbacks can be effectively avoided by using an advanced detection framework—rather than human or traditional radar detection—to build an anti-UAV system [6,7,8]. The main focus of current research is to enhance the learning frequency and quality of the detection model for small targets compared with larger targets to improve performance in such cases. However, a problem that is overlooked is that the design of the convolutional neural network itself leads to information loss for low-resolution targets. Meanwhile, most existing anti-UAV detection datasets contain only samples of UAVs and not other types of flying objects, such as birds, that are visually similar to UAVs from the perspective of detection systems. Hence, when such an object appears in the image, it can easily be mistakenly detected as a UAV. In other words, the anti-interference ability of the detection algorithm remains insufficient, raising concerns about its robustness in real-world scenarios [9].

To address data scarcity, we built a clean, reliable, and sample-balanced anti-interference anti-UAV detection dataset containing four types of flying objects for training the anti-interference ability of the detector. In addition to UAVs, it also includes aircraft, helicopters, and birds, providing comprehensive support for the anti-interference training of the anti-UAV detector to ensure that it avoids falsely identifying these flying objects as UAVs.

In this study, we develop an anti-interference anti-UAV detection method based on the YOLOv9-C model to improve the detection of small UAV targets. Dot distance is used as an evaluation index to assign positive and negative samples during the training stage. As a result, the number of positive samples of the small UAV target is increased, improving detector performance in the case of small targets. The comparison of experiment results on the proposed dataset demonstrates that the developed method achieves better mean average precision than other methods.

## 2. Related Works

### 2.1. Anti-UAV Detection Algorithm in Infrared Scenes

Detection of UAVs using infrared images has attracted interest as a technology based on thermal imaging that allows effective UAV detection even in low-light conditions, such as those encountered at night. Many methods have been proposed for detecting targets in infrared scenes, and traditional examples include the local-contrast-based method WLLCM [10] and the filtering-based method top-hat [11]. However, these handcrafted feature-based approaches struggle to adapt to the complex variations in real-world scenarios. With the increasing availability of computational resources, deep learning-based methods have become mainstream. DNANet [12], for instance, employs a densely nested network to reinforce the deep feature representation of infrared targets for more precise detection, while IRGraphSeg [13] uses a graph neural network to facilitate powerful attention, allowing modeling of more complex and variable detection environments.

Although using infrared-based anti-UAV detection technology allows a detector to overcome its reliance on lighting conditions, it still faces significant drawbacks under natural light. First, since any object above absolute zero can emit infrared radiation, various elements in the image may trigger false positives in detection algorithms. Second, infrared target boundaries are often blurry and lack color and texture information. Third, targets are typically represented by fewer pixels using infrared imagery compared to natural light. These factors pose substantial challenges for detectors in learning and accurately identifying targets.

In summary, while infrared anti-UAV detection technology has distinct advantages, using regular images remains a more effective approach in well-lit environments.

### 2.2. Anti-UAV Detection Algorithm Based on Deep Learning

Currently, most existing deep learning-based anti-UAV detection algorithms can be divided into three categories: those in the first use high-resolution features of UAV; those in the second use attention mechanisms to suppress clutter in the background; and those in the last one increase the sample size of small objects. Each of these is described in detail below.

Cui et al. [2] proposed a UAV detection algorithm combining an improved YOLOv3 model and super-resolution reconstruction technology. First, potential regions where the target may exist are identified. Super-resolution reconstruction is then applied to these regions to add detailed information. Finally, a dimensional clustering method is used to regenerate the model pre-selection parameters, adjusting their allocations for high-precision anti-UAV detection. Yang et al. [3] developed a rough-to-fine computational architecture that, first, calculates the rough locations of UAVs with low-level features and then gradually refines them using sparse convolution. In this way, high-resolution features can be obtained while minimizing the computation of the background.

To address the challenge of insufficient texture information for small objects, Yang et al. [5] introduced an attention mechanism, dividing it into global channel attention, local channel attention, and spatial attention. At the same time, a small anchor frame was adapted to match the size of small objects, and more accurate detection results were obtained. Li [7] effectively enhanced the contrast by incorporating information on the differences between the region of interest and surrounding areas using top-hat. Using this approach as a basis, a target enhancement method based on difference statistics was proposed to make small targets more visually significant. Ge [8] improved the structure of feature pyramid networks in YOLOv3 by proposing an adaptive feature fusion method that combines the coordinate structure of channel attention and location information attention to enhance the mean average precision (mAP).

Using a training dataset comprising a large number of simulated images and a small number of real images, Wang et al. [14] solved the problem of cross-domain adaptation by using feature transfer, increasing the mAP for sea-sky scenes to 94.21%. To address the issues of non-obvious features and challenging extraction of small targets, Xu et al. [15] designed an IoU prediction module based on the RefineDet network to enhance the localization of small targets and developed a target moving algorithm for indirectly increasing the number of small targets, effectively avoiding their missed detection.

To summarize, the main focus of current research is to increase the learning frequency and quality of the detection model for small targets compared with other scale targets. Although the model has improved performance on small targets, a problem that is overlooked is that the convolutional neural network itself will lead to information loss for low-resolution targets. This limitation makes it challenging to consistently achieve satisfactory results, as important details needed for detecting small targets may be lost during the process.

### 2.3. Anti-UAV Detection Dataset

The dataset is the foundation of deep learning, playing a crucial role in the validation and evaluation of models. In actual anti-UAV detection environments, there are not only UAVs exhibiting various movements but also other visually similar flying objects such as birds, planes, and helicopters. For example, a helicopter and a UAV may likely appear to have the same size and shape when the helicopter is far away from the detector while the UAV is close. Therefore, to enhance detection performance, anti-UAV detection algorithms need to not only be able to successfully detect UAVs under various conditions but also accurately capture the fine-grained differences, thereby avoiding confusion between UAVs and other flying objects. At present, most known anti-UAV detection datasets contain only UAV targets and lack other types of flying objects, and there are very few datasets dedicated to training anti-UAV detectors in anti-interference capabilities.

Table 1 lists several open-source datasets used for anti-UAV detection. Paweczyk et al. [16] captured images of UAVs in flight from YouTube videos and constructed an anti-UAV detection dataset based on a top-down perspective, named Real World, which includes multiple types of UAVs. However, the scale of UAV targets is too large to effectively simulate the actual working scenes of anti-UAV detectors. Zheng et al. [17] constructed the anti-UAV detection dataset Det-Fly by using a UAV to photograph other UAVs in flight, including upward, downward, and oblique views, to overcome the shortcomings of UAV data where there is only a single viewing angle. However, the dataset contains only one type of target, with no other types of flying objects, which limits its usefulness for training the anti-interference capability of anti-UAV detectors. Similarly, this is also a problem for the MIDGRAD [18] and USC-Drone [19] datasets.

Table 2 introduces some open-source datasets containing multiple classes used for anti-UAV detection. To train anti-UAV detectors, Kashiyama et al. [20] built a high-resolution flight object dataset FOD by using 4K resolution cameras to photograph flying objects. Although there are three classes of targets in the dataset—birds; aircraft; and helicopters—UAVs are unfortunately not included. The IEEE International Conference on Advanced Video and Signal-based Surveillance (AVSS) released a drone-bird dataset, named DVB-AVSS, at the 2021 Anti-Drone Challenge [21]. The IEEE International Conference on Acoustics, Speech, and Signal Processing (ASSP) also released a drone-bird dataset, named DVB-ASSP, at the 2023 Anti-Drone Challenge [22]. Both datasets contain UAVs and birds and can therefore be used to train detectors to correctly detect UAVs in the presence of bird interference. Despite the large size of these two datasets, they contain only one type of disturbance: birds.

To summarize, it is urgent that other types of flying objects be introduced in building a more comprehensive anti-UAV dataset, so as to improve the training of anti-UAV detectors in handling a broader range of interference scenarios.

## 3. The Anti-Interference and Anti-UAV Detection Dataset

To enhance the anti-interference ability of UAV detectors, we introduced multiple classes of flying objects in constructing an anti-interference and anti-UAV dataset, named Anti2, which has a total of 5062 images. In addition to UAV targets, Anti2 also contains three other different types of flying objects: planes, helicopters, and birds, and therefore presents a greater challenge for the anti-interference ability of the anti-UAV detector. The images in the Anti2 were sourced in the following four ways:

(1) Collecting from open-source object detection datasets, including the single-class anti-UAV datasets Real World [16] and Det-Fly [17], the bird detection dataset SOD4SB [23], the helicopter detection dataset HelicoptersofDC [24], and the plane detection dataset PD [25].

(2) Collecting from open-source object-tracking datasets, including the UAV target-tracking datasets Anti-UAV [26], BVD [21], and DroneDetection [27].

(3) Extracting from video sites, including Bilibili and YouTube.

(4) Acquiring from image search engines, including the text search engines Google, Bing, and Baidu, and the image search engines Yandex and TinEye.

**Figure 1:**
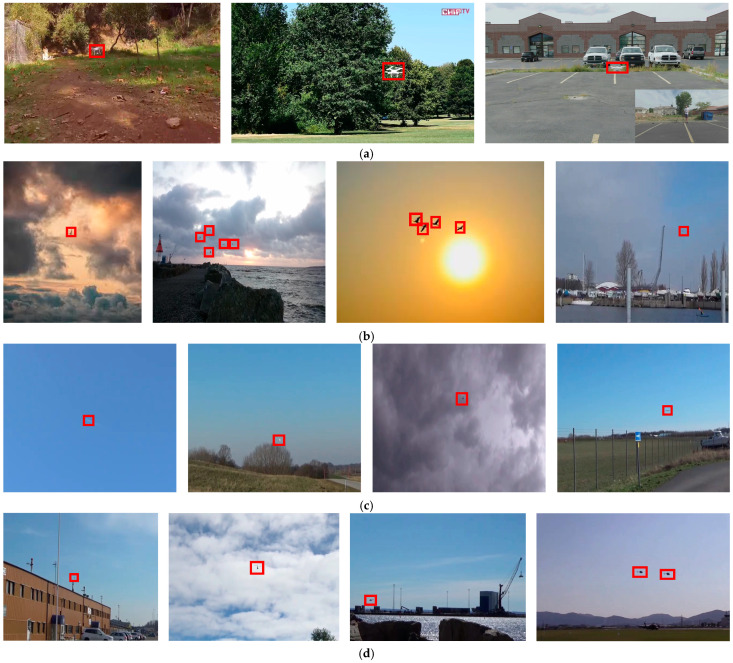
Example images of the Anti2 dataset. (**a**) UAV targets; (**b**) Bird targets; (**c**) Airplane targets; (**d**) Helicopter targets.

The image annotation tool “LabelImg” is used to mark the bounding boxes for the four classes of flying objects in the Anti2 dataset, and the annotation information was saved as a txt file in YOLO format, which can be adapted to all YOLO series models. A total of 5828 boundary boxes of flying targets were identified, including 1688 UAV targets, 1444 bird targets, 1406 helicopter targets, and 1320 airplane targets. Some example images of the Anti2 dataset are shown in Figure 1. We can clearly see that most objects are in flight and their scale in the picture is small, which indicates that the proposed dataset contains images that closely reflect the actual deployment scenarios for anti-UAV detection.

**Figure 2:**
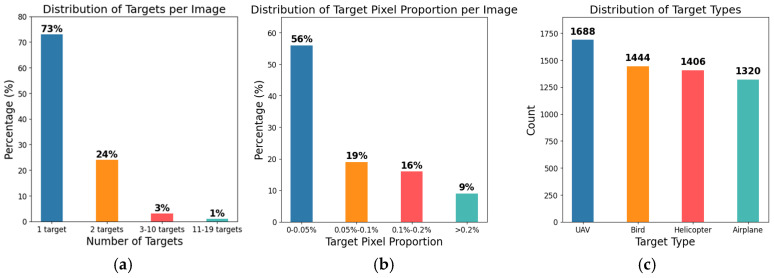
Diversity of targets in the Anti2 dataset. (**a**) Targets per image; (**b**) Target pixel proportion; (**c**) Target types.

**Figure 3:**
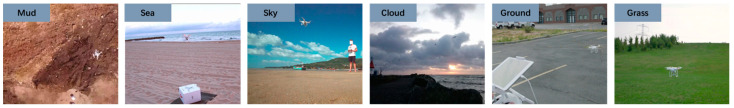
Diversified background of the Anti2 dataset.

To train the detector to adapt to different operational environments, our dataset includes images with varying backgrounds, such as mud, sea, sky, cloud, ground, and grass, as shown in Figure 2 and Figure 3. Additionally, to more closely match actual anti-UAV scenarios, our dataset encompasses both single- and multi-target cases, with the target sizes being generally small.

## 4. The Proposed Anti-UAV Detection Algorithm

To prevent the detector from confusing UAV targets with other types of flying objects while simultaneously considering UAV targets at an extremely small scale, we used YOLOv9-C [28] as the baseline model and selected dot distance (DotD) [29] as the evaluation index to construct an anti-UAV detection algorithm for natural light scenes, referred to as DotD-YOLOv9-C.

### 4.1. The Introduction to YOLOv9

YOLOv9 is an anchor-free, end-to-end object detection framework [28] in which a series of improvements to YOLOv7 are introduced to achieve a top-tier level of detection. Firstly, the generalized efficient layer aggregation network (GELAN) is proposed as an extension of the efficient layer aggregation network (ELAN) [30]. Secondly, a reparameterization technique is used to make GELAN lightweight while maintaining high reasoning speed and accuracy. Secondly, the ELAN module in YOLOv7 is replaced with GELAN, and five versions of YOLOv9 with varying parameter numbers (T/S/M/C/E) are designed [28]. Finally, to accelerate the convergence speed and improve the model’s robustness, a programmable gradient information (PGI)-assisted monitoring framework is proposed. Compared to earlier YOLO series models, the most distinctive improvements in YOLOv9 are the generalized high-efficiency layer aggregation network and PGI-assisted monitoring framework.

#### 4.1.1. The Generalized Efficient Layer Aggregation Network

Most existing network design strategies are based on the feed-forward path, where the network architecture is structured according to the data path, which can effectively extract features of certain physical significance. However, this strategy often results in parameters at different layers learning the same knowledge during back propagation, which reduces their utilization. To address this issue, Wang et al. [31] proposed a network architecture design based on gradient paths, which can allow some channels to be connected across stages and differentiate the learned gradients by different layers, thereby reducing computational overhead and improving the efficiency of parameter use. 

The multi-branch convolution part of ELAN is generalized based on the gradient path design strategy, which allows it to be replaced with multiple arbitrary compute modules. This generalized network framework is called GELAN, which enables reducing the number of calculations of these modules and efficient parameter use to achieve enhanced performance. Moreover, by using a reparameterization technique, GELAN can utilize a multi-branch architecture to complete training while reassembling the trained parameters into a single-branch architecture for inference, which greatly improves the inference speed of YOLOv9.

#### 4.1.2. The Programmable Gradient Information

In deep neural networks, the transformation of data across multiple layers can lead to accumulating information loss. According to the information bottleneck principle, as the number of network layers increases, the model will not be able to fully retain the predicted information about the target, which will cause the network to use unreliable gradients in the training process, which ultimately complicates the convergence process. To solve this problem, programmable gradient information (PGI) can be utilized to train the network. PGI acts as an auxiliary branch that only participates in computation during training and provides the original network with a reliable gradient; during inference, this branch can be directly removed without affecting the inference time. When YOLOv9 is trained using PGI, the convergence rate significantly improves. Moreover, the loss function of the auxiliary branch can be customized to improve YOLOv9 performance in the direction expected by users, which greatly increases its flexibility and adaptability.

(1) Auxiliary reversible branch

The problem of unreliable gradients, which is encountered when the number of layers of neural networks is deepened, is addressed using reversible architecture (i.e., using a network comprising reversible functions for training), which ensures that the loss function generates reliable gradients during back propagation. However, directly applying a reversible architecture to the YOLOv9 backbone increases inference costs. Therefore, the reversible architecture can be designed as an auxiliary branch of the original model that is outside the YOLOv9 backbone network. During the inference stage, the auxiliary reversible branch can be directly removed without consuming any additional computational resources. During the training stage, the auxiliary reversible branch receives the same input as the main model and independently calculates losses to generate a reliable gradient. These gradients will promote parameter learning of the backbone network, help extract correct and important information, and enable the backbone model to obtain more efficient properties for the target task without increasing inference costs.

(2) Multilevel auxiliary information

Multilevel auxiliary information refers to the insertion of an integrated network between the main branch and the feature pyramid level of the auxiliary branch. This integrated network is responsible for first aggregating the gradients returned from different prediction heads and then transferring them to the master branch to update parameters. By doing so, each stage of the master branch can learn the gradient information of different scale targets, thereby improving the auxiliary effect of PGI for training the backbone network.

### 4.2. The Introduction to Dot Distance

In the task of anti-UAV detection, UAVs typically appear far away from the detector, resulting in very small scales within the image. Intersection over union (IoU) is the most popular evaluation index for common target detection tasks, but it exhibits excessive sensitivity to position in the case of very small targets, making it less suitable for training anti-UAV detectors. With the development of target detection technology, some new indexes have successively emerged, such as GIoU [32], DIoU [33], and CIoU [34], but their performance in detecting small UAV targets remains unsatisfactory. To address this issue, Xu et al. [29] proposed a simple yet effective evaluation index, dot distance (DotD), defined as the index of normalized Euclidean distance between the center points of two boundary boxes, which is more suitable for anti-UAV detection tasks where small targets are prevalent and accurate position evaluation is crucial.

The calculation process of DotD is as follows:

(1) For all targets of the same class in the dataset, their average size *S* is calculated using the following equation:

(1) $$S=\sqrt{\frac{\sum_{i=1}^{M}\sum_{j=1}^{N_{i}}w_{ij}h_{ij}}{\sum_{i=1}^{M}N_{i}}}$$

where *M* represents the total number of images, and *N_i_* represents the number of bounding boxes in the *i*th image.

(2) For detecting bounding box *A* and labeling bounding box *B*, the Euclidean distance between their center points *D* is calculated as

(2) $$D=\sqrt{{\left(x_{A}-x_{B}\right)}^{2}+{\left(y_{A}-y_{B}\right)}^{2}}$$

where $\left(x_{A},y_{A}\right)$ and $\left(x_{B},y_{B}\right)$ represent the coordinates of the center points of each of the two bounding boxes.

(3) Normalizing the Euclidean distance between the center points of the two bounding boxes and calculating the DotD as

(3) $$DotD=e^{-\frac{D}{S}}$$

Compared to other indexes, DotD is more effective in detecting small targets, with a more reasonable strategy for allocating positive and negative samples, making it better suited for anti-UAV detection tasks.

### 4.3. The Proposed DotD-YOLOv9-C Detection Algorithm

**Figure 4:**
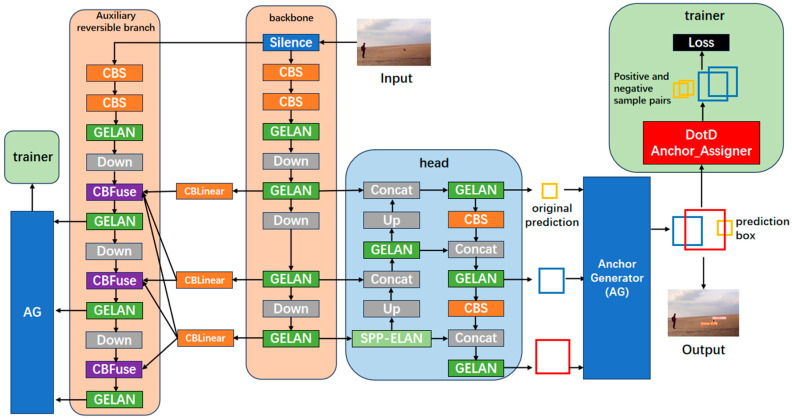
The network structure of DotD-YOLOv9-C.

To improve the detector performance on small targets, we construct the detection algorithm DotD-YOLOv9-C. In this approach, YOLOv9-C is utilized as the baseline model, and the small-target-friendly evaluation index DotD is used to allocate positive and negative samples. The network structure is shown in Figure 4.

Based on the information processing flow, the main branch of the model can be divided into three primary modules: the backbone, the head, and the trainer. Specifically, during the training process, an additional auxiliary reversible branch with similar structure is connected to the main branch via CBLinear and participates in the computation, providing more effective supervisory information to the main branch, and then is omitted during inference. 

In the backbone, the model first performs feature extraction on the input and transmits feature maps of different scales to the head. At the entry, two layers of CBS—comprising convolution; batch normalization; and the SiLU activation function—use large kernels for pre-downsampling the input. This is followed by consecutive convolution and downsampling, where convolution is implemented through ELAN, which can be summarized as follows:

(4) $$M_{i+1}=CB_{4}\left[P_{I},P_{II},P_{III},P_{IV}\right]$$

(5) $$P_{I}=CB_{1}^{I}(M_{i})$$

(6) $$P_{II}=CB_{1}^{II}(M_{i})$$

(7) $$P_{III}=CB_{2}^{I}(CB_{1}^{III}(M_{i}))$$

(8) $$P_{IV}=CB_{3}^{I}(CB_{2}^{II}(CB_{1}^{IV}(M_{i})))$$

where *M_i_* denotes the feature map in stage *i*, *P_i_* denotes the portion *i* of channels in the feature map, [ ·, ·] denotes the concatenation layer, and *CB_i_^j^*(·) represents the portion *j* of the output of the computational block *i*. The computational block can be any necessary convolution module, which refers specifically to RepNCSP and CBS in YOLOv9-C. ELANs multilayer half-channel skip connections significantly enrich the gradient paths at the output nodes, increase the combinations of different channels, and enhance the model’s expressive capacity.

After receiving feature maps of various scales, the head employs a combination of upsampling and downsampling to fuse the features and then outputs the raw prediction data. The SPP-ELAN module, a version of the SPP module that has been improved based on the gradient path combination concept, enables the feature maps to be fused in multiple ways.

The auxiliary reversible branch also follows an information processing flow similar to that of the main branch. First, an identical input is provided to the auxiliary reversible branch for computation. Along the way, multi-scale feature maps calculated by the backbone of the main branch are duplicated and sent to the auxiliary reversible branch for feature fusion. After predictions are generated, the loss is computed with the same loss function in the main branch. During backpropagation, the supervisory signal is transmitted back to the main branch through CBLinear, contributing to the optimization of the backbone parameters and enhancing the overall performance of the model. It is worth noting that if the task requires the model to acquire specific capabilities through training, the loss function in the auxiliary reversible branch can be replaced with any function designed to meet these particular requirements.

The trainer is responsible for providing training supervision signals, active only when training. When detection boxes produced by the anchor generator arrive in the trainer, the inner anchor assigner is required to sort and match these detection boxes with the labels according to the selected evaluation index. Once the predictions are assigned, the loss is computed and backpropagated to optimize the trained model. The quality of the positive and negative samples allocated in this way will directly affect the final training result. In other words, the evaluation index will influence the strategy for allocating positive and negative samples, thereby affecting the quality of model training. However, sample-assigning evaluation indexes such as IoU, GIoU, DIoU, and CIoU exhibit high position sensitivity and are not suited to small-scale detection boxes. Although these detection boxes better match UAV targets in scale, their assignment as positive samples remains challenging because, under the influence of these evaluation indexes, small-scale detection boxes cannot score higher than slightly large-scale detection boxes even with the same positioning accuracy, thus ranking lower in the anchor assigner. Therefore, the strategy for positive and negative sample allocation generated using these evaluation indexes will, to a certain extent, hinder model learning regarding the detection of small-scale targets, ultimately reducing the performance of anti-UAV target detectors.

To address the weakness associated with IoU, DotD was introduced as the evaluation metric in YOLOv9-C, resulting in the development of a high-precision anti-UAV detector, DotD-YOLOv9-C. As shown in Figure 4, prior to the prediction boxes being assigned as positive and negative training samples, they are scored by DotD instead of IoU. In predicting targets with extremely small scales, DotD evaluates based on Euclidean distance, ensuring that small prediction boxes are not mistakenly considered as further from the target than the larger boxes. As a result, the targets can be correctly allocated as positive samples. This shows that using DotD to score prediction boxes is an effective strategy for preventing high-precision prediction boxes from receiving low scores due to their smaller scales. Therefore, DotD can be used instead of IoU in the anchor assigning phase to provide a higher-quality positive sample frame for training, thereby improving the quality of model training and, consequently, the performance of the anti-UAV target detector.

## 5. Experiment Analysis

### 5.1. Experimental Setup

In this work, all the experiments were performed in the hardware and software configurations listed in Table 3.

For all experiments involving DotD-YOLO-C and its baseline model, the input image size was 512 × 512, and all the images in Anti2 were randomly divided into the training, verification, and test sets according to a 6:2:2 ratio, corresponding to 3038 images, 1012 images, and 1012 images, respectively. Moreover, due to video memory limitations, the batch size for model training was set to 2, and the epoch to 300. We initialized the model using the pre-trained weights officially provided in [28] and used the SGD optimizer with a momentum coefficient of 0.937 for parameter optimization. The first three epochs were set for the warm-up phase, in which the learning rate gradually increased from 0.0012 to 0.003 (i.e., the initial learning rate), and the momentum gradually increased from 0.8 to 0.937. In the following training process, the OneCycleLR scheduling strategy was used to dynamically adjust the learning rate, which increases from 0.003 to a higher value and then gradually decreases back to 0.0003.

### 5.2. Evaluation Index

In this work, the detection performance of different models was compared based on the metrics precision, recall, average precision, and mean average precision.

*Precision* is the fraction of relevant instances among the retrieved instances, written as a formula:

(9) $$Precision=\frac{TP}{TP+FP}$$

*Recall* is the fraction of relevant instances that were retrieved, written as a formula:

(10) $$Recall=\frac{TP}{TP+FN}$$

where *TP*, *FP*, and *FN* represent the number of true positive, false positive, and false negative samples, respectively. In this work, the threshold for IoU is set to 0.5.

Average precision is used to evaluate the detection precision for each category and is defined as

(11) $$AP_{i}=\int_{0}^{1}P(R)dR$$

where *AP_i_* denotes the average precision of the *i*th category, and the Anti2 dataset includes four categories: UAV, bird, airplane, and helicopter.

Moreover, mean average precision is used to evaluate the overall performance of the detector. It is defined below:

(12) $$mAP=\frac{1}{C}\sum_{i=1}^{C}AP_{i}$$

where *C* denotes the number of categories, which is 4 in this work.

### 5.3. Comparison and Visualization of Experiment Results

Experimental verification was executed to quantify the enhancement from using the DotD index in anti-UAV detection training. We separately applied GIoU, DIoU, CIoU, and DotD to the baseline model for training, yielding the experimental results shown in Table 4. The results indicate that using DotD for training in this task not only accelerates convergence but also improves the overall performance of the detector.

**Figure 5:**
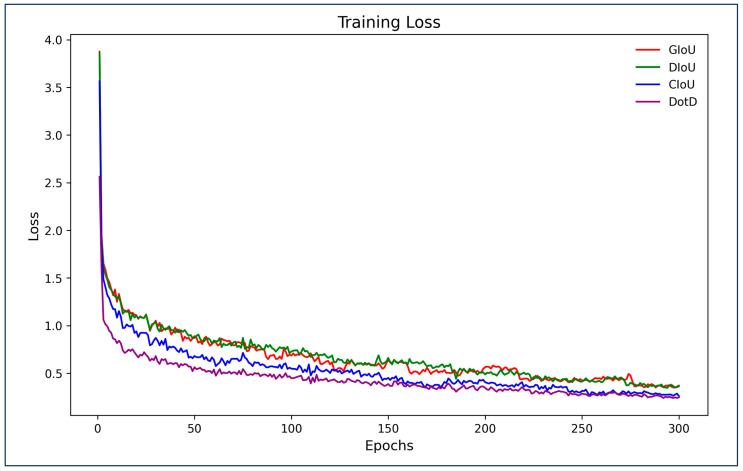
Training loss of YOLOv9-C trained with different indexes.

**Figure 6:**
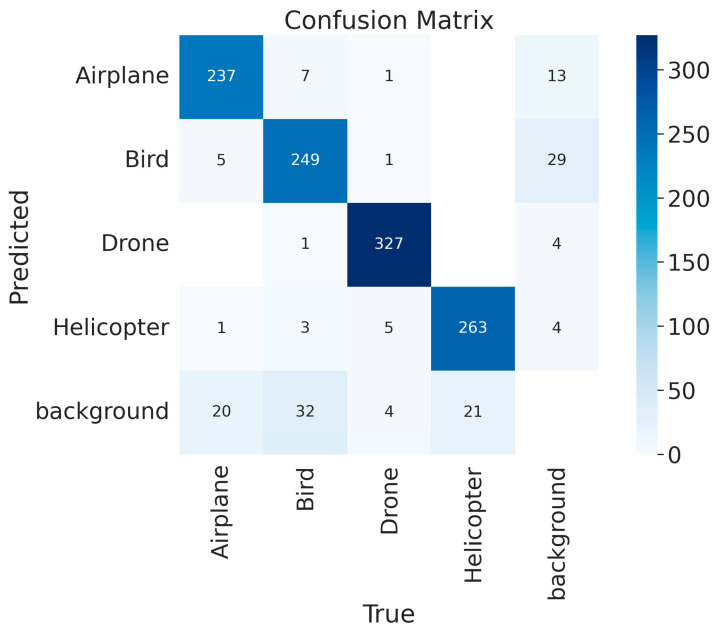
Confusion matrix of DotD-YOLOv9-C.

To verify the effectiveness of the proposed anti-UAV detection method, we conducted an experiment on the Anti2 dataset involving comparison with other methods. In this comparative experiment, all the methods presented were configured according to the descriptions in the original papers, including the models, loss functions, hyperparameters, etc. All models were trained and validated using the train and validation sets of Anti2. These uniformly trained models were then tested on the Anti2 test set, and the experimental results are presented in Table 5. The training process is shown in Figure 5, and the confusion matrix of the proposed method is shown in Figure 6.

**Figure 7:**
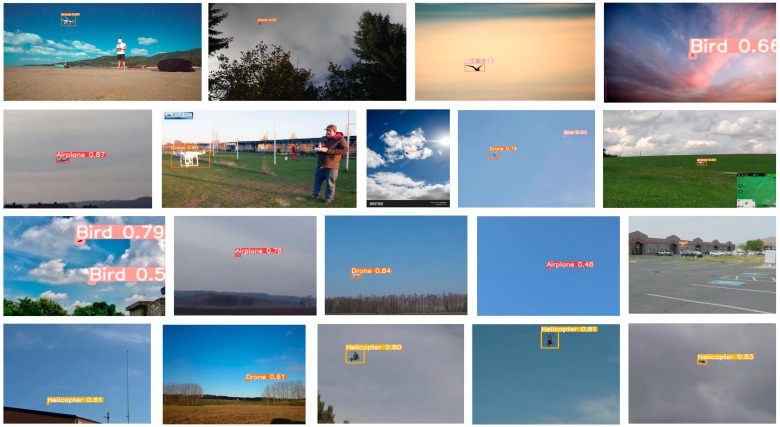
Some correct detection results of DotD-YOLOv9-C.

The experiment results show a mAP of 92.6% for DotD-YOLOv9-C, which outperforms the other methods used in the comparison. Some examples of anti-UAV detection are shown in Figure 7. Compared to the baseline method, the biggest accuracy improvement of DotD-YOLOv9-C is achieved for the bird class, which has the most small-scale target and multi-target images. To some extent, this indicates that the positive and negative sample allocation strategy based on DotD helps the model better adapt to small-scale targets, thereby improving performance in the actual anti-UAV detection environments.

**Figure 8:**
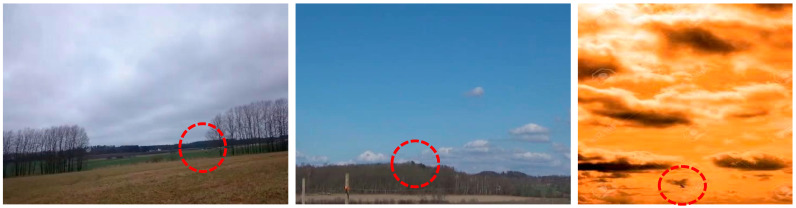
Some mistake detection results of DotD-YOLOv9-C.

In most cases, our proposed anti-interference anti-UAV detection method, DotD-YOLOv9-C, can correctly detect UAV targets with high AP values. However, some detection failures remain for the Anti2 dataset, primarily involving missed targets. As illustrated in Figure 8, in the left image, the UAV resembles a distant house in shape; in the middle image, the white UAV is hidden against a cloud; in the right image, the birds appear black during dusk and have a similar shade to the clouds. All of these scenarios can lead to missed detection using the anti-UAV detection method. The possible causes of these errors may include the following: (1) the target has a similar shape to other non-flying objects; (2) the target color is very similar to the background color. These problems suggest that the dataset should be enriched with more images where the target and background have a similar color. In addition, the detection of non-flying objects with similar shapes should be considered, as both factors will help minimize missed detections by the detector. We will also continue to study anti-UAV detection under various lighting conditions and weather states to further improve the method’s robustness in complex environments. Additionally, although this method enhances the model’s ability to learn small targets by reducing the location sensitivity of the metric, its effectiveness in preventing information loss for small targets in the deeper layers of the network remains limited. In the future, we may explore network structures designed to strengthen feature representation in these deeper layers.

## 6. Conclusions

To improve the anti-interference capability of anti-UAV detectors, we constructed a dataset containing four types of flying objects: UAVs, birds, airplanes, and helicopters. This dataset comprises 5062 images, with a total of 5828 targets labeled by bounding boxes. The dataset can be used to train a detector to better distinguish UAVs from birds, airplanes, and helicopters. Moreover, an anti-interference anti-UAV detection method was proposed for detecting small UAV targets in which YOLOv9-C is used as the baseline model and dot distance as the evaluation index for assigning positive and negative samples. A comparison of experiment results on the Anti2 dataset shows that our method achieves a higher mean average precision than other methods, indicating the effectiveness of the dataset and detection approach. 

In the future, we may collect and add other types of flying objects into the anti-interference anti-UAV detection dataset for further improvement. We will also investigate anti-UAV detection under different lighting and weather conditions. Additionally, we may explore methods to enhance feature representation in deeper layers.

## Figures and Tables

**Table 1:** Statistics from open source datasets containing single class Dataset Class Size Resolution Label.

Dataset	Class	Size	Resolution	Label
Real World [12]	UAV	56,821	640 × 640	bounding box
Det-Fly [13]	UAV	13,271	3840 × 2160	bounding box
MIDGARD [14]	UAV	8775	752 × 480	bounding box
USC-Drone [15]	UAV	18,778	1920 × 1080	bounding box

**Table 2:** Statistics from open source datasets containing multiple classes.

Dataset	Class	Size	Resolution	Label
FOD [16]	helicopter/birds/plane	1630	3840 × 2160	bounding box
DVB-AVSS [17]	UAV/birds	106,568	720 × 576	bounding box
DVB-ASSP [18]	UAV/birds	106,568	3840 × 2160	bounding box

**Table 3:** Experimental environment.

Equipment	Setup
Operating System	Ubuntu 18.04
CPU	AMD Ryzen 9 7945HX with RAdeon Graphics
GPU	NVIDA GeForce RTX 4060 Laptop GPU, 16G NVIDIA, Santa Clara, CA, USA.
GPU Acceleration	CUDA 12.3, cuDNN 8.9.6
Programming Language	Python 3.8.19
Framework	Pytorch 2.3.0
Editor	Anaconda 23.11.0

**Table 4:** mAP, FPR, and FNR values achieved by YOLOv9-C trained under different indexes.

Indexes	Categories				mAP	FPR	FNR
	UAV	Bird	Helicopter	Airplane			
GIoU	96.3%	80.6%	93.5%	86.0%	90.3%	2.5%	5.4%
DIoU	96.8%	85.0%	92.5%	84.2%	90.7%	2.2%	4.6%
CIoU	97.6%	84.0%	95.0%	86.1%	91.4%	0.9%	4.2%
DotD	97.8%	87.1%	94.2%	91.4%	92.6%	1.6%	3.1%

**Table 5:** The Average Precision of different methods in each category.

Methods	Categories				mAP
	UAV	Bird	Helicopter	Airplane	
Querydet [6]	95.3%	72.4%	91.7%	84.8%	86.0%
DETR [35]	97.4%	82.9%	93.2%	87.7%	90.3%
YOLOv9-C [29]	97.6%	84.0%	95.0%	86.1%	90.7%
GFL [36]	98.1%	82.4%	95.6%	89.5%	91.4%
DotD-YOLOv9-C	97.8%	87.1%	94.2%	91.4%	92.6%

## Data Availability

The proposed dataset is available at https://github.com/gdpinntit/-anti-interference-and-anti-UAV-dataset.
